# Familial intellectual disability as a result of a derivative chromosome 22 originating from a balanced translocation (3;22) in a four generation family

**DOI:** 10.1186/s13039-017-0349-x

**Published:** 2018-02-20

**Authors:** Zhang Kaihui, Huang Yan, Dong Rui, Yang Yali, Wang Ying, Zhang Haiyan, Zhang Yufeng, Gai Zhongtao, Liu Yi

**Affiliations:** 1 Pediatric Research Institute, Qilu Children’s Hospital of Shandong University, 23976 Jingshi Road, Jinan, Shandong 250022 China; 2 Rehabilitation Center, Qilu Children’s Hospital of Shandong University, 23976 Jingshi Road, Jinan, Shandong 250022 China

**Keywords:** 3q duplication syndrome, 22q13.3 microdeletion syndrome, Balanced translocation

## Abstract

**Background:**

Balanced reciprocal translocation is usually an exchange of two terminal segments from different chromosomes without phenotypic effect on the carrier while leading to increased risk of generating unbalanced gametes. Here we describe a four-generation family in Shandong province of China with at least three patients sharing severe intellectual disability and developmental delay resulting from a derivative chromosome 22 originating from a balanced translocation (3;22) involving chromosomes 3q28q29 and 22q13.3.

**Methods:**

The proband and his relatives were detected by using karyotyping, chromosome microarray analysis, fluorescent in situ hybridization and real-time qPCR.

**Results:**

The proband, a 17 month-old boy, presented with severe intellectual disability, developmental delay, specific facial features and special posture of hands. Pedigree analysis showed that there were at least three affected patients. The proband and other two living patients manifested similar phenotypes and were identified to have identically abnormal cytogenetic result with an unbalanced translocation of 9.0 Mb duplication at 3q28q29 and a 1.7Mb microdeletion at 22q13.3 by karyotyping and chromosome microarray analysis. His father and other five relatives had a balanced translocation of 3q and 22q. Fluorescence in situ hybridization and real-time qPCR definitely validated the results.

**Conclusions:**

The abnormal phenotypes of the proband and his two living members in four generations of the family confirmed the 3q duplication and 22q13.3 deletion inherited from familial balanced translocation. This is the first report of familial balanced reciprocal translocation involving chromosomes 3q28q29 and 22q13.3 segregating through four generations.

## Background

Balanced reciprocal translocation, the most common chromosomal rearrangement in humans, is usually an exchange of two terminal segments from different chromosomes without genetic material loss which occur in 0.16%–0.20% (1/625–1/500) of live births [1–3]. Almost all balanced translocation have no phenotypic effect on the carrier but lead to increased risk of generating unbalanced gametes. Of the significant hazards is unfavorable pregnancy outcomes such as recurrent miscarriages, still births, early newborn deaths, or the offspring with birth defects due to the different forms of the unbalanced gametes produced during the meiotic segregation of chromosomes. Meiotic segregation of reciprocal translocation produces gametes with a variety of combinations of normal and translocated chromosomes. The partial chromosome complement of 32 possible zygotes could be produced by the union of gametes from a translocation carrier parent and a non-carrier parent [4].

Here, we describe a four-generation Chinese family with six individuals carrying a karyotypically balanced chromosomal translocation t(3;22)(q28;q13) manifesting normal phenotype, while there are three patients with severe intellectual disability and developmental delay carrying 3q28q29 duplication and 22q13.33 deletion. The conventional cytogenetic analysis combined with chromosome microarray identified the submicroscopic imbalances deciphering the etiology of such patients in the family. We reviewed the literature of partial trisomy 3q associated with 3q duplication syndrome [5] and 22q13.3 microdeletion syndrome [6], and discussed the genotype-phenotype correlation related to this case.

## Methods

### Clinical description

**Fig. 1:**
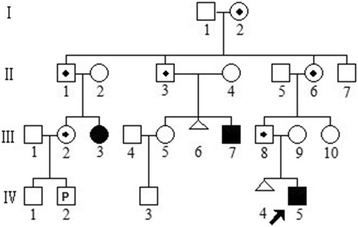
The pedigree chart of the family. The pedigree chart of the family. The proband and additional two relatives are affected patients. His father and five members are obligate carriers. His mother is normal. □ healthy male; ○ healthy female; ■/●male/female patient; /male/female obligate carrier;  proband;  dead;  abortion;  pregnancy;  not detected

The proband, 17 month-old boy, is the first child of a couple of unrelated healthy parents. His mother had one time of active abortion before (see Fig. 1 for the pedigree chart of the family) without obviously inducing factors. The boy’s gestation period was normal.

The boy was born at 40 weeks of gestation with weight of 3.2 kg and length of 50 cm. His head circumference was 34 cm and Apgar score was 10. His audition test was normal. The boy showed normal development at birth. From 6 month-old, he gradually displayed developmental retardation. At age of 17 month, his mental and motor development was significantly delayed. He was 12 kg mass and 80 cm tall, the head circumference was 44 cm. He showed facial dysmorphism with protruding forehead, bushy eyebrows, big eyes, hypertelorism, big cup ears, low nasal bridge, downturned corners of the mouth, pointed jaw, and apathia, and special posture of hands including both hands ulnar deviation at the state of relation, middle finger straight while other fingers bending, strong proximal belly-finger, and grasping only with his thumb and middle finger. His development was tested with Gesell Developmental Observation-Revised (GDO-R) demonstrating extremely severe developmental delay for adaptability (score: 22 points), moderate delay for gross motor (score: 41 points) and fine motor (score: 51 points), severe delay for language (score: 38 points) and personal-social interaction (score: 36 points). Now, He could mumbled “mama, baba” in occasional unconsciousness, but neither walked nor followed instructions. He manifested less eye contact with casual eye tracking. Other examinations including magnetic resonance imaging of brain, electroencephalogram (EEG), cardiac and abdominal ultrasound were all normal.

A total of 22 family members in four generations were investigated in the family. Additional two family members (III:3 and III:7) were found to manifest the same phenotypes as the proband. The oldest living patient (III:7) was a 21-year-old male presenting severe intellectual disability, speech disorder, motor retardation, specific facial features and special posture of hands.

### G-banding karyotyping

Peripheral blood leukocytes from the proband and other family members were stimulated by phytohemagglutinin. Routine cytogenetic analysis by G-banding techniques at the 400 bands of resolution was performed using imaging software for humans according to the International System for Human Cytogenetic Nomenclature (ISCN, 2016).

### Fluorescence in situ hybridization (FISH) analysis

To verify the balanced translocation found in karyotyping and to validate the obligate carriers in the family, subtelomeric FISH studies were performed using Agilent SureFISH probes (Agilent, Beijing, China) for 3p26.2 (SureFISH 3p26.2 CNTN4 RD, Orange Red) and 3q29 (SureFISH, 3q29 WDR53 211 kb, Green), 22q13.33 (SureFISH 22q13.33 SHANK3, Green) and 22CEP (SureFISH 22CEP, Orange Red) according to manufacturer’s procedure. Selected subtelomeric probes were used for carriers and healthy family members to identify balanced carriers. The 22CEP probe is not a centromere specific but a locus specific centromere-near probe at 22q11.

### Chromosome microarray analysis (CMA)

Chromosome microarray was performed for the proband using Affymetrix CytoScan HD array (Affymetrix, Santa Clara, CA), and data were analyzed with the software of Chromosome Analysis Suite (ChAS) (Affymetrix, Santa Clara, CA) using the following filtering criteria: deletions > 5 kb (a minimum of five markers) and duplications > 10 kb (a minimum of 10 markers). DNA digestion, ligation, fragmentation, labeling, hybridization, staining and scanning were performed following the Affymetrix’s protocol. The Database of Genomic Variants (GRCh37/hg19) and OMIM, DECIPHER, ISCA were used to evaluated the array data and analyze genotype-phenotype correlation.

### Real-time quantitative PCR validation

To verify the chr22q13.3 microdeletions in the patients of the family, a pair of primers were designed to target the deleted gene SHANK3 (chr22:50674415-50733298) using an online primer designing tool-Primer 3 (http://primer3.ut.ee/) and synthesized by Shanghai Invitrogen Biotechnology Company (Shanghai, China). Assays were carried out in accordance with manufacturer recommendations on the 7500 Real-Time PCR system (Applied Biosystems, Foster city, California). The copy number variations were determined based on the ratio of deletion fragment copies to reference gene (GAPDH) copies in samples. Both genomic DNA samples from the normal male and female individuals were used simultaneously as two control samples. Each qPCR was carried out in triplicate with the SYBR Premix Ex Taq II PCR reagent kit (TakaRa Bio, Dalian, China) according to the manufacturer’s protocol.

## Results

### Karyotyping

**Fig. 2:**
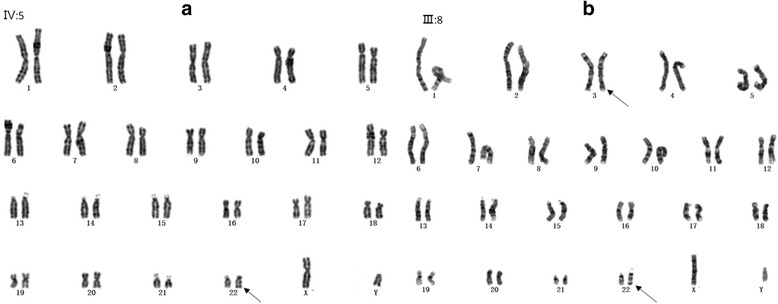
Representative G-banding karyotyping results. **a** Representative karyotype of the patient (proband IV:5) showing der(22)t(3;22). **b** Representative karyotype of obligate carriers (the proband’s father, III:8) showing t(3;22)(q28;q13)

Fifty metaphase cells were examined for the proband and other family members. An apparently abnormal karyotype was identified in proband (IV:5) and two living relatives (III:3 and III:7) as der(22)t(3;22) (q28;q13.3). The derivative chromosome in proband (IV:5) was paternally (III:8) inherited, whereas his mother (III:9) showed a normal karyotype. In other family members, the apparently balanced t(3;22) (q28;q13.3) translocation was present in six living persons of I:2, II:1, II:3, II:6, III:2 and III:8. The representative results are shown in Fig. 2a, b.

### FISH

**Fig. 3:**
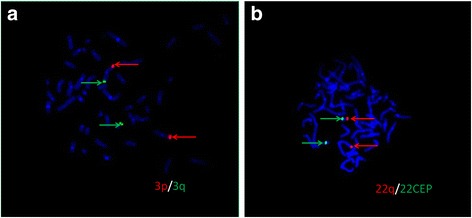
**a** Representative results from the carrier (proband’s father III:8) showing 3q29 signal (Green) separated from 3p26 signal (Red); **b** Representative results from the carrier (proband’s father III:8) showing 22q13.3 signal (Red) separated from 22 CEP (a locus specific centromere-near probe) signal

FISH was performed for predicted carriers and healthy members of the family. The results showed that 22q13.33 signal (Red) separated from 22CEP signal and translocated into 3q in proband’s father (III:8) and other five members of I:2, II:1, II:3, II:6 and III:2, indicating that the six family members were obligate carriers of balanced translocation of 3q and 22q (Fig. 3a, b). The abnormal unbalanced translocation in proband was inherited from his balanced translocation carrier father.

### CMA

**Fig. 4:**
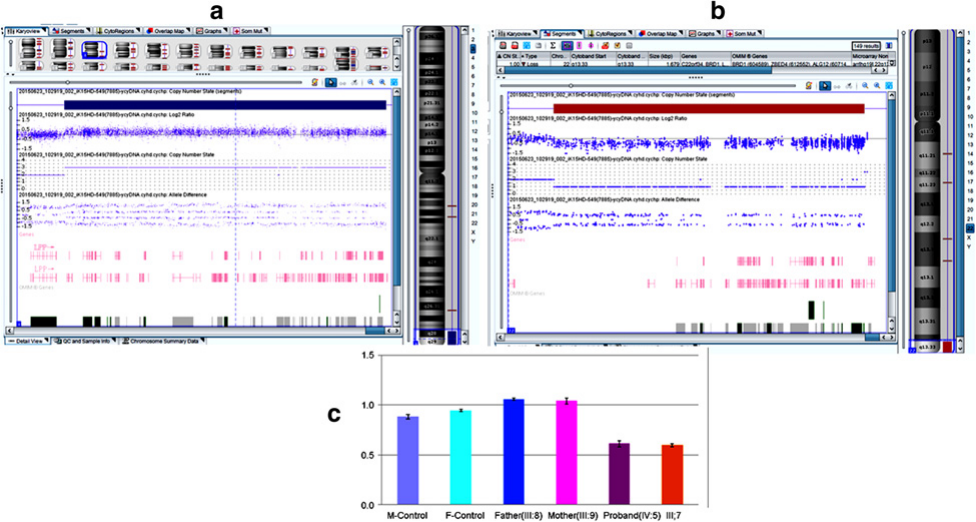
CMA and real-time qPCR results. Whole-genome Chromosome microarray analysis for the proband(IV:5) was arr[GRCh37]. 3q28q29(188823885–197,851,986) × 3,22q13.33(49,505,152–51,183,871) × 1 pat. **a** CMA showing a 9.0-Mb duplication at 3q28q29; **b** CMA showing a 1.7-Mb deletion at 22q13.33. **c** Real-time qPCR showing the proband(IV:5) and the living patient (III:7) had the deletion of *SHANK3* gene in 22q13.33, but the proband parents (III:8, III:9) were normal as control

To detect the microdeletion in 22q13.33 and identify exact variation of genes involved in the abnormal chromosome of the patients. CMA was carried out and two abnormal copy number variants were identified as follows: arr[GRCh37] 3q28q29(188823885-197851986)× 3,22q13.33(49,505,152-51,183,871) × 1 pat. It included a 3q terminal duplication (9.0 Mb) and a 22q terminal deletion (1.7 Mb) (Fig. 4a, b). Terminal 3q duplication contains lots of genes, the important OMIM genes like CLDN1 and CLDN16, are partially overlapped with a previously described region related to 3q duplication syndrome. Terminal 22q deletion contains 38 RefSeq genes (having 27 OMIM genes and a critical SHANK3 gene) associated with 22q13.3 microdeletion syndrome (Phelan-McDermid syndrome, PMS, OMIM: 606230).

### Real-time quantitative PCR

Real-time quantitative PCR performed on the family and two control samples (both male and female healthy individuals) showed that the proband and the living patient (III:7) had the deletion of SHANK3 gene in 22q13.33 and the proband’s parents were same as controls (Fig. 4c).

## Discussion

Intellectual disability (ID) is a common, variable and heterogeneous manifestation of central nervous system dysfunctions affecting 1–3% of the population [6]. Unfortunately only less than one-half of ID could be identified the specific etiologies [7, 8]. In this study, we described a familial balanced reciprocal translocation involving chromosomes 3q and 22q segregating through four generations in which six family members (I:2, II:1, II:3, II:6, III:2 and III:8) carrying the balanced translocation. The proband was firstly referred to our hospital for his severe physical and mental retardation, and then at least three patients with the same phenotypes were found in his four-generation family by means of the pedigree investigation which highlighted the potential chromosomal abnormalities in the family. By using the G-banding karyotyping analysis, an abnormal long arm of charomosome 22 was found in the proband. To study the etiology of the proband, CMA was applied and showed the derivative chromosome 22 with 9.0 Mb duplication of 3q28q29 and 1.7 Mb microdeletion of the 22q13.33.

It is known that the subtelomeric regions among the gene rich regions of the genome, are particularly prone to recombination and therefore often involved in chromosomal rearrangements, in which subtle rearrangements at the telomere regions may cause unexplained ID [9], and many of these subtelomeric deletions or duplications are now recognized as clinically recognizable phenotypes [9, 10]. According to the literature, the 3q26.3-3q29 duplications being the minimal critical region could cause 3q duplication syndrome [11], and the 22q13.33 microdeletion was associated with 22q13.3 deletion syndrome (Phelan-McDermid syndrome, PMS) [12].

Arıkan et al. [13] summarized the most common abnormal features of 3q duplication syndrome, such as facial dysmorphism (hypertrichosis, prominent eyelashes, bushy eyebrows, broad nose with anteverted nares and depressed nasal bridge hypertelorism, epicanthic folds, long philtrum, micrognathia, low anterior hairline, malformed auricles), limb anomalies (rhizomelic shortening of the limbs, hypoplasia of the phalanges, camptodactyly and clinodactyly), congenital heart defects (septal defects), renal malformations (polycystic kidneys or dysplasia), seizures, brain malformations, and so on. However, partial trisomy 3q cases with duplication of different segments showed significant differences from each other. Approximately 60–75% cases with 3q distal duplication have a concomitant deletion of another chromosomal segment carrying unbalanced translocation, and the deletion of the chromosomal segment could contribute to the phenotype as the present case [14, 15]. Our case has characteristic features of the 3q duplication syndrome such as facial features (protruding forehead, bushy eyebrows, hypertrichosis, low nasal bridge, and malformed auricles), special posture of hands (camptodactyly and clinodactyly) and the severe development delay (mental, motor, and language).

It has been reported that the EPHB3, CLDN1 and CLDN16 located at 3q26.31–q29 were important to 3q duplication syndrome [11]. Our case has a 9.0-Mb duplication of 3q28q29 encompassing CLDN1 and CLDN16. CLDN1 (OMIM 603718) and CLDN16 (OMIM 603959) locating at 3q28 encode Claudin 1 and Claudin 16, respectively, which are epithelial or endothelial cell-to-cell adhesion tight junction proteins. Loss of Claudin 1 function mutations could result in neonatal ichthyosis-sclerosing cholangitis syndrome [16, 17], while the gene mutations in CLDN16 could cause familial hypomagnesemia with hypercalciuria and nephrocalcinosis (FHHNC) which is a rare autosomal recessive renal disease [18]. The detailed mechanism of how haploinsufficiency of CLDN1 and CLDN16 causing disorders remains to be elucidated. It has been known that the fragment duplication of 3q28 encompassing CLDN16 was associated with multiple congenital abnormalities including coarctation of the aorta, atrial septal defect (ASD) and ventrical septal defect (VSD), hypertrichosis and umbilical hernia/omphalocele [19], while our patients presented the phenotype of hypertrichosis in which CLDN16 might play an important role.

In addition, the present case was identified to have a 1.7 Mb 22q subtelomeric deletion associated with 22q13.3 deletion syndrome also known as Phelan-McDermid syndrome (PMS) [20]. PMS is characterized by developmental delay, absent or impaired speech, neonatal hypotonia, autistic traits and mild dysmorphic features. Shank3 encoding by SHANK3 gene (also known as PROSAP2, OMIM: 606230) is a postsynaptic scaffolding protein with the key role in spine shape/maturation, localization of glutamate receptors, and growth cone motility [21]. The mutations of SHANK3 gene have been considered to be responsible for the neurological features of the PMS phenotype [22]. The association between deletion size and phenotypes expanded the genomic region of interest in PMS, in which small deletion size is mainly related to autism spectrum disorders, but large deletion size is prone to severe phenotypes. In the DECIPHER database, all deletions referred to PMS ranging in size from 100 kb to over 9 Mb contain SHANK3, and 75% PMS cases carried the simple terminal deletions while approximately 25% cases were comprised of translocations in the 22q13 region, ring chromosome 22 and mosaics [23, 24]. Our case had a 1.7 Mb deletion including 38 RefSeq genes and his family patients were accompanied by PMS phenotypes: severe developmental delay, hypotonia, speech/language delay, facial dysmorphism. We deduced that the variations of large fragment and genes referring to unbalanced translocation of 3q and 22q contributed to the severe phenotypes.

## Conclusions

In this study, we presented the molecular cytogenetic characterization of 3q28q29 duplication and 22q13.33 microdeletion in a proband with severe intellectual disability and developmental delay. This is the first report of a familial reciprocal translocation t(3,22)(q28;q13.3) segregating through four generations. Three patients carrying an unbalanced condition with der(22)t(3:22) were found, It was reported that a cross-like quadrivalent configuration in gametocytes of reciprocal translocation carriers was usually observed [3, 25]. The meiotic segregation patterns of the quadrivalent are alternate, adjacent-1, adjacent-2 and 3:1. We deduced the unbalanced state in the patients was likely from an adjacent-1 segregation, in which der(22)t(3:22) was rearranged. Perhaps, there is another possibility that the chromosomal pairs 3 and 22 were arranged in bivalents instead of quadrivalents (as usual for a translocation) in meiosis. However, homologous chromosomes for up to 9.0 Mb of 3q Subtelomere failed to pair together during meiosis, which is a great hazard for the long unmatched fragment in meiosis. Meanwhile, most researches showed that the actual proportion of normal and balanced translocation zygotes for the offspring of reciprocal translocation carriers was much higher than the theory of 2:32. Also the interesting fact that up to now only male carriers of the balanced translocation produced an offspring with der (22) in the family. All of the above need to be further validated by the pairing configurations occurred in pachytene substage. We reviewed the literature of 3q duplication syndrome and 22q13.3 microdeletion syndrome, and discussed the genotype–phenotype correlation in this case, suggesting that prevention of recurrent intellectual disability in this family can be achieved through carrier screening and prenatal genetic diagnosis.

## Abbreviations

ASD: Atrial septal defect
CMA: Chromosome microarray
FHHNC: Familial hypomagnesemia with hypercalciuria and nephrocalcinosis
FISH: Fluorescence in situ hybridization
ID: Intellectual disability
PMS: Phelan-McDermid syndrome
VSD: Ventrical septal defect
