# Correction to: Familial intellectual disability as a result of a derivative chromosome 22 originating from a balanced translocation (3;22) in a four generation family

**DOI:** 10.1186/s13039-018-0373-5

**Published:** 2018-04-23

**Authors:** Kaihui Zhang, Yan Huang, Rui Dong, Yali Yang, Ying Wang, Haiyan Zhang, Yufeng Zhang, Zhongtao Gai, Yi Liu

**Affiliations:** 1 Pediatric Research Institute, Qilu Children’s Hospital of Shandong University, 23976 Jingshi Road, Jinan, 250022 Shandong China; 2 Rehabilitation Center, Qilu Children’s Hospital of Shandong University, 23976 Jingshi Road, Jinan, 250022 Shandong China

## Correction

In the original publication [1] the author names were in the wrong order. The correct version can be found in this Correction. The original article has been updated to rectify this error.

Incorrect version:

Zhang Kaihui, Huang Yan, Dong Rui , Yang Yali, Wang Ying , Zhang Haiyan , Zhang Yufeng , Gai Zhongtao and Liu Yi

Correct version:

Kaihui Zhang, Yan Huang, Rui Dong, Yali Yang, Ying Wang, Haiyan Zhang, Yufeng Zhang, Zhongtao Gai and Yi Liu.
